# Observation of gold sub-nanocluster nucleation within a crystalline protein cage

**DOI:** 10.1038/ncomms14820

**Published:** 2017-03-16

**Authors:** Basudev Maity, Satoshi Abe, Takafumi Ueno

**Affiliations:** 1 School of Life Science and Technology, Tokyo Institute of Technology, 4259-B55, Nagatsuta-cho, Midori-ku, Yokohama 226-8501, Japan

## Abstract

Protein scaffolds provide unique metal coordination environments that promote biomineralization processes. It is expected that protein scaffolds can be developed to prepare inorganic nanomaterials with important biomedical and material applications. Despite many promising applications, it remains challenging to elucidate the detailed mechanisms of formation of metal nanoparticles in protein environments. In the present work, we describe a crystalline protein cage constructed by crosslinking treatment of a single crystal of apo-ferritin for structural characterization of the formation of sub-nanocluster with reduction reaction. The crystal structure analysis shows the gradual movement of the Au ions towards the centre of the three-fold symmetric channels of the protein cage to form a sub-nanocluster with accompanying significant conformational changes of the amino-acid residues bound to Au ions during the process. These results contribute to our understanding of metal core formation as well as interactions of the metal core with the protein environment.

## Abstract (web summary)

Proteins can template the synthesis of inorganic nanoparticles, but the formation mechanisms remain vague. Here, the authors directly observe, through a sequence of X-ray crystal structures, the stages of gold sub-nanocluster growth within the confined environment of a ferritin cage.

The use of protein scaffolds as biotemplates for synthesis of uniform inorganic nanoparticles has attracted significant attention because such nanoparticles can be applied as catalysts, imaging reagents and magnetic materials in bionanotechnology^1^,^2^,^3^,^4^,^5^,^6^,^7^. Nanoparticles with various shapes (including spherical and tubular nanoparticles, for example) can be synthesized by taking advantage of the unique structures of protein assemblies. It is believed that metal nanoparticles in protein scaffolds are formed by deposition of metals, followed by nucleation, which leads to the formation of a small sub-nanocluster as the metal core. The sub-nanocluster metal core will then grow by aggregation to form the nanoparticles^8^. Thus, it is essential to investigate the formation of sub-nanoclusters in protein scaffolds to gain an understanding of the molecular mechanisms of biomineralization and protein-nanoparticle recognition^8^,^9^. There are only a few reports of X-ray crystal structures of metal oxide/chloride nanostructures such as Fe_*x*_O_*y*_, W/MoO_*x*_ and Cd_7_Cl_12_, which are formed within the symmetric cavities of protein scaffolds^10^,^11^,^12^,^13^,^14^. These reports show that the symmetrical environments of protein scaffolds can be used for nanocluster formation. However, the dynamic process for the growth mechanism of metallic nanoclusters with metals such as Au, Ag, Pd and so on in protein environments remains unclear due to the lack of structural information.

To gain insights into the molecular mechanism of metal nanoparticle growth in a protein scaffold, it is essential to structurally characterize the process of metal ion deposition within a protein scaffold and the initial formation of the core metal nanocluster. Theil^15^ studied the translocation of Fe ions into the ferritin cage by X-ray crystallography. Recently, deposition and characterization of various metal ions into protein scaffolds has been reported^9^,^16^. The process of nucleation of metal ions to form nanoclusters in protein environments has also been studied by spectroscopy and microscopy^17^,^18^. However, the structural information about the process of nanoparticle formation is still too lacking to understand the mechanism. It therefore remains challenging to determine the structure of a sub-nanocluster in a protein scaffold as an intermediate structure of nanoparticle growth in order to understand the mechanism of its formation.

In the efforts to determine the structure of a sub-nanocluster in a protein environment, metal ions can be chemically reacted inside the protein crystals and X-ray structures can be obtained at different stages to understand the process of nanoparticle formation. Chemical reactions promoted inside single crystals can trap reaction intermediates, which provides direct evidence of the reaction mechanism^19^,^20^,^21^,^22^. Protein crystals have recently attracted significant attention as protein-assembly materials^9^,^23^. Protein crystals have unique porous structures comparable to typical porous materials and can be increased in mechanical strength by crosslinking, which is known as one of the most convenient techniques in protein crystal engineering^23^. Thus, the crosslinked crystals, which maintain their lattice structures and three-dimensional protein structures, are suitable for studying chemical reactions under various conditions by X-ray crystal structure analysis. So, it will be interesting to promote the chemical reduction of Au ions inside a single crystal of protein template for determination of the X-ray crystal structures at different intermediate stages to understand the process of formation of the Au sub-nanocluster in a protein environment.

Among various protein templates, the self-assembled 24-mer protein cage of ferritin is of particular interest as it is naturally designed for iron storage. Iron translocation and biomineralization inside the ferritin cage are studied extensively^10^,^24^,^25^,^26^,^27^,^28^. Besides the natural functions, the spherical apo-ferritin cage has been widely used as a template for preparing homogeneous or uniform non-natural metal nanoparticles that have important biomedical applications^29^,^30^,^31^. Due to rapidly growing interests in ferritin and other protein-templated nanomaterials, there is an increasing demand for understanding the fundamentals of nanoparticle growth in protein environment, which is important for rational design and fine-tuning of their properties. Therefore, we chose the ferritin protein cage as a model protein template for the study. However, the formation process of non-natural metal nanoparticles inside the ferritin cage and their interactions with protein environment remain unclear due to lack of crystal structures. Previously, deposition of silver ions into the ferritin cage followed by nanoparticle formation was studied by Oksana *et al*.^32^. The growth mechanism of cobalt oxide nanoparticles in the ferritin cage was studied by transmission electron microscopy, which showed the initial formation of small metal cores at the interior wall of the ferritin cage^18^. The structural details for the accumulation of Pd ions or Pd(allyl) complexes into the ferritin cage were also reported^16^,^33^. The structures indicated that the three-fold symmetric channels of the cage are used for accumulation of a number of metal ions^16^,^26^,^34^,^35^. It is expected that such pre-organized metal ions in the narrow channel of ferritin can be converted to a single sub-nanocluster by reduction reaction because the unique coordination environment in the confined protein cavity stabilizes the intermediate structures with flexible amino-acid residues, as previously described in the literatures^10^,^12^. However, the reduction reaction in ferritin crystal, which is different from metal coordination reactions previously observed^16^,^33^, has not been achieved because the ferritin crystals decomposed under such harsh reduction conditions.

Herein we describe a crystal structure analysis of Au(III) ions accumulated in the ferritin cage and subsequent reduction of the metal ions by sodium borohydride inside the crosslinked single crystal. Significant results of the work include direct observation of formation of the sub-nanocluster at the symmetric three-fold axis channel accompanied by conformational changes of amino-acid residues. Movement of Au ions is deduced from the crystal structures at different intermediate steps.

## Results

### Molecular design of the crystalline protein cage

**Figure 1:**
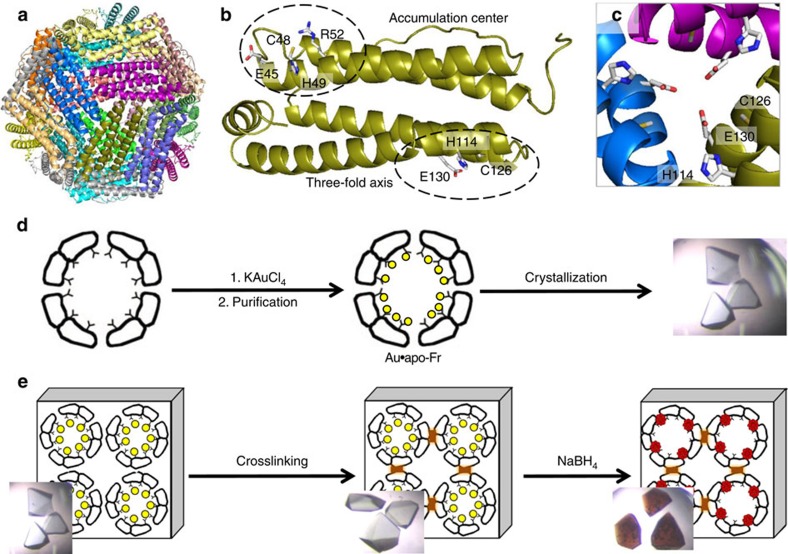
Structure, metal immobilization and chemical reaction inside a single crystal of L-ferritin. (**a**) Whole structure of 24-mer assembly (PDB ID 1DAT). (**b**) Monomeric subunit showing the positions of specific metal binding region and (**c**) view of a symmetric three-fold axis channel. (**d**) Reaction scheme for the preparation of Au·apo-Fr and crystallization. (**e**) Schematic representation for the crosslink treatment and reduction of Au ions inside the crosslinked single crystal.

Previous reports on metal-ferritin composites show that Fr (ferritin) has two specific metal binding regions, which are located at the three-fold axis channel and the accumulation centre (Fig. 1a–c)^16^,^36^. The metal accumulation site of the apo-Fr (wild type) is centred at Cys48 and surrounded by His49, Glu45 and Arg52. Since Au ions have high affinity for sulfur, it was expected that introduction of additional Cys residue would enhance uptake of Au ions into the cage. Using this concept, we replaced the Glu45 and Arg52 residues of recombinant L-chain apo-Fr from horse liver (apo-rHLFr) by Cys and the resulting apo-rHLFr mutant apo-E45C/R52C-rHLFr was used in the current study. Incorporation of Au(III) ions into the apo-E45C/R52C-rHLFr cage was achieved by stirring KAuCl_4_ (200 equiv) with apo-E45C/R52C-rHLFr in 0.15 M NaCl (pH 8.5), followed by dialysis against 0.15 M NaCl and gel filtration (Sephadex G-25) (Fig. 1d). The results of inductively coupled plasma mass spectrometry (ICP-MS) measurements and bicinchoninic acid assay (BCA) analyses indicate the presence of a total of 107±4 Au atoms per apo-E45C/R52C-rHLFr cage.

**Figure 2:**
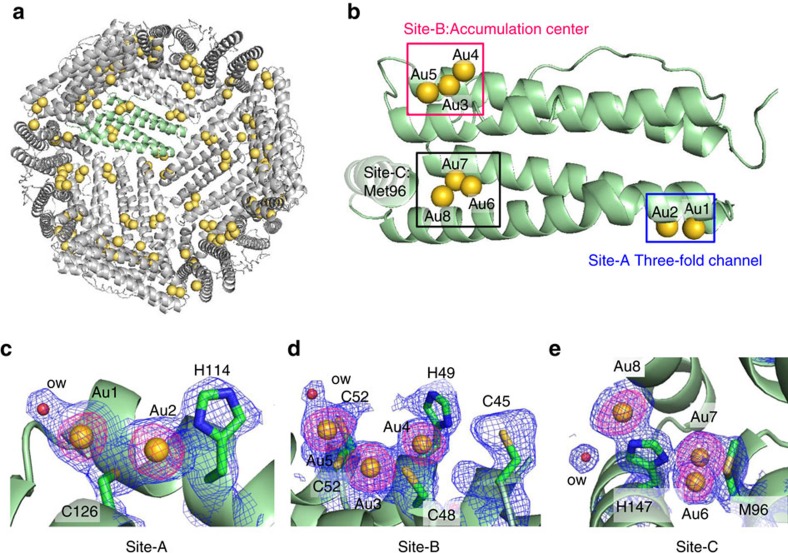
Crystal structure of Au·CL-apo-E45C/R52C-rHLFr. (**a**) Whole structure showing the positions of Au ions immobilized on the interior surface. (**b**) Monomeric subunit showing the binding sites of Au ions. (**c**–**e**) Coordination structures of Au at three-fold channel (Site-A), accumulation centre (Site-B) and Met96 (Site-C) binding regions. The Au atoms are shown as yellow spheres. Water molecules are shown as red spheres. 2*F*_o_-*F*_c_ electron density maps at 1*σ* are shown in blue and anomalous difference Fourier density maps at 4*σ* are shown in pink.

The Au accumulated ferritin cage (Au·apo-E45C/R52C-rHLFr) was crystallized using the hanging drop vapour diffusion method with (NH_4_)_2_SO_4_/CdSO_4_ as the precipitant, as required for ferritin crystallization^36^. The single crystals were reinforced by crosslinkage treatment in glutaraldehyde solution to maintain the lattice structure for structural analysis of the chemical reaction within the crystal (Fig. 1e). The structure of the crosslinked crystal (Au·CL-apo-E45C/R52C-rHLFr) was refined to 1.95 Å resolution. Selected crystallographic parameters are provided in the supporting information (Supplementary Table 1). The root-mean-square deviation of the C_α_ atoms of Au·CL-apo-E45C/R52C-rHLFr from that of apo-rHLFr is 0.25 Å. This indicates that the folding of apo-rHLFr is preserved even after the accumulation of Au(III) ions and the crosslink treatment. The positions of the Au atoms were assigned from the anomalous difference Fourier density map. The Au atoms are distinguished from Cd by comparison with the control ferritin structure, anomalous density maps at Au peak (1.038 Å) and remote (1.058 Å) wavelengths, and previous reports^36^,^37^. Although the total number of Au binding sites observed from the crosslinked crystal is 192, the sum of Au atoms estimated from all of the occupancies is 96, which is consistent with that obtained from the quantitative analyses (ICP/BCA) (107±4) (Supplementary Table 2). This indicates that the Au atoms adopt several coordination modes and therefore all of the Au atoms are superimposed on one monomer subunit of the crystal structure with low occupancy of electron density, as described in previous reports^16^,^24^,^33^,^36^,^37^. All of the Au binding sites in the ferritin cage are divided into three deposition regions; the three-fold axis channel (Site-A), the metal accumulation site (Site-B) and at Met96 (Site-C) (Fig. 2a,b). At Site-A, there are two Au atoms, one of which (Au1) is coordinated by Cys126 and a water molecule (Fig. 2c). The other Au ion (Au2) at Site-A is coordinated by both His114 and Cys126. Cys126 forms a bridge between Au1 and Au2, giving Au–S^γ^(Cys) distances of 2.39 and 2.26 Å, respectively. The linear geometry suggests the possible +1 oxidation state of the Au ions^37^. At Site-B, three different Au ions (Au3–Au5) are present, which form a thiol-bridged (Cys48 and Cys52) trinuclear structure (Fig. 2d). His49 weakly interacts with Au5, forming an Au5–N^ɛ^ distance of 2.74 Å. The observed Au–S distances are in the range of 2.19–2.31 Å (Supplementary Table 3). At Site-C, Au6 and Au7 are coordinated by His147 and Met96 (Fig. 2e). The close distance (2.35 Å) between Au6 and Au7 suggests that they do not exist simultaneously. Au8 is coordinated only by His147. The observed Au–N^ɛ^(His147) distances are in the range of 2.15–2.44 Å, which is within the typical Au–N distance range^38^.

### Chemical reaction within the crosslinked ferritin crystal

**Figure 3:**
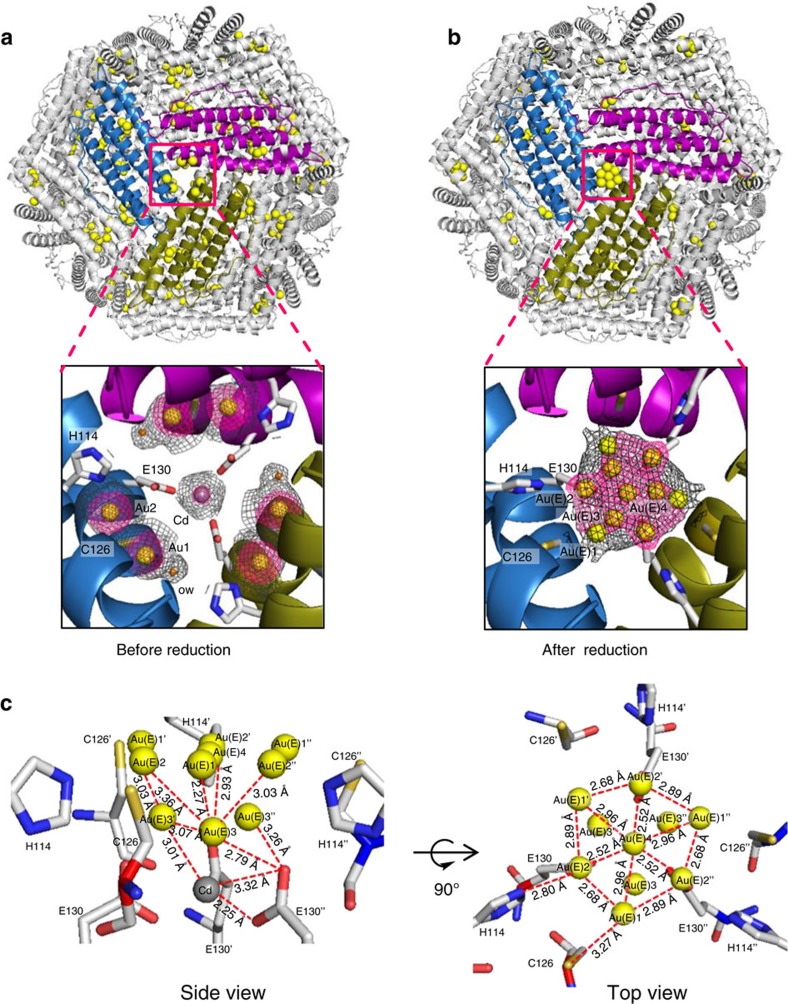
Crystal structures showing the changes of Au atoms after the reaction with NaBH_4_. (**a**) Crystal structure of Au·CL-apo-E45C/R52C-rHLFr showing the pre-organization of Au atoms at the three-fold channel. (**b**) Crystal structure of Au^0^(E)·CL-apo-E45C/R52C-rHLFr showing the formation of Au sub-nanocluster at the three-fold channel after the reduction of pre-organized Au atoms in Au·CL-apo-E45C/R52C-rHLFr with NaBH_4_. (**c**) Binding site of the Au sub-nanocluster formed in Au^0^(E)·CL-apo-E45C/R52C-rHLFr with selected bond distances. The side view is showing the two-layered structure of the sub-nanocluster and the interactions of the lower layer with the adjacent Au atoms from upper layer, Cd atom and Glu130. The top view is showing the interactions of the upper layer of the sub-nanocluster with adjacent His114 and Cys126. The Au and Cd atoms are shown as yellow and grey spheres, respectively. The selected 2*F*_o_*-F*_c_ maps at 1*σ* and anomalous difference Fourier density maps at 4*σ* are shown in grey and pink colours, respectively.

After determining the binding positions of Au ions inside the ferritin cage, we became interested in observing the changes of the positions of Au ions when reduced with sodium borohydride (NaBH_4_) within the single crystal. We performed the reduction reaction by soaking the crosslinked single crystals (Au·CL-apo-E45C/R52C-rHLFr) into a solution containing excess (250 mM) NaBH_4_ for 1 h. The resulting composite is represented as Au^0^(E)·CL-apo-E45C/R52C-rHLFr (Fig. 1e). During the reaction, the colour of the crystals changed to dark brown, indicating reduction of Au ions (Fig. 1e)^9^. The absorption spectrum of the Au^0^(E)·CL-apo-E45C/R52C-rHLFr crystals showed a weak absorption near 550 nm, which could be assigned to surface plasmon absorbance of the small nanocluster formed inside the protein cage (Supplementary Fig. 1)^39^. In order to determine the structure of the nanocluster, we analysed the X-ray crystal structure of Au^0^(E)·CL-apo-E45C/R52C-rHLFr, which was refined to 2.03 Å resolution (Fig. 3b). Selected crystallographic parameters are included in the supporting information (Supplementary Table 4). The root-mean-square deviation of the C_α_ atoms from apo-rHLFr is 0.21 Å, which indicates that the overall spherical cage structure of the apo-rHLFr cage is preserved even after the reduction reaction of Au ions inside the protein crystal. This shows the importance of crosslink treatment for structural studies on chemical reaction within protein crystal. The crystal structure analysis of Au^0^(E)·CL-apo-E45C/R52C-rHLFr shows a large anomalous density at the three-fold symmetric channel of the ferritin cage, which was absent before the reduction reaction (Fig. 3a,b). This clearly indicates the formation of Au nanoclusters at the three-fold axis channels. Since the outer and inner diameter of the three-fold channel is 8.5 and 5.4 Å, respectively, the observed Au nanoclusters have sub-nanometer size, which fits into the channels^40^. X-ray diffraction measurements at Au peak (1.035 Å) and remote (1.057 Å) wavelengths confirmed that the cluster is formed by Au atoms with a single Cd atom, which is essential for ferritin crystallization (Supplementary Fig. 2). The Cd atom stabilizes the Au sub-nanocluster by forming the Au_3_Cd unit as described previously (Supplementary Fig. 3)^41^,^42^,^43^. The Au clustering site at the three-fold channel was modelled based on the observed anomalous difference Fourier density map (Fig. 3c) and the Au atoms were refined with partial occupancy (Supplementary Table 5). The Au-Au bond distances in the Au clustering site are observed in the range of 2.52–3.07 Å. These bond distances are similar to bond distances reported in the literature^41^,^42^,^44^. The observed Au clustering site has two layers (Fig. 3c). The His114 residue from three different monomers stabilizes the upper layer of the Au cluster, giving an Au(E)2–N^ɛ^(His114) distance of 2.80 Å (Fig. 3c). No stabilizing ligand was observed near the exterior of the three-fold channel. The bottom layer of the sub-nanocluster, which is directed towards the internal cage, consists of three Au atoms stabilized by a Cd atom and three Glu130 residues, giving Au(E)3–O^δ^(Glu130) distances of 2.79 Å (Fig. 3c). The observed Au(E)3–Cd distance is 3.01 Å, which is consistent with the previous literature reports^41^,^42^. The Au sub-nanocluster formed in the symmetric channel of the protein cage is unique in nature and different from Au nanoclusters stabilized by organic ligands^45^,^46^. Therefore the unique arrangement of the Au atoms in the three-fold channel appears to be reinforced by the symmetric protein environment. As observed for the three-fold channel, changes in the positions of Au atoms are observed at other Au binding sites (Supplementary Fig. 4). In the metal accumulation centre, the Au4 coordinated by the alternate conformer of Cys52 disappears and new anomalous electron densities are observed near His49 (Supplementary Fig. 4a). This indicates another possible clustering site near His49, but no atoms were assigned due to insufficient electron density. The positions of Au3 and Au5 coordinated by Cys48 remain unchanged. The Au atoms at binding Site-C (Met96) disappear (Supplementary Fig. 4b) and no regions of similar electron density are observed nearby. Therefore, the crystal structure analysis of Au^0^(E)·CL-apo-E45C/R52C-rHLFr suggests that there are two Au clustering sites in the apo-E45C/R52C-rHLFr cage that are located at the three-fold channel and the metal accumulation site.

### Process of formation of the Au sub-nanocluster

**Figure 4:**
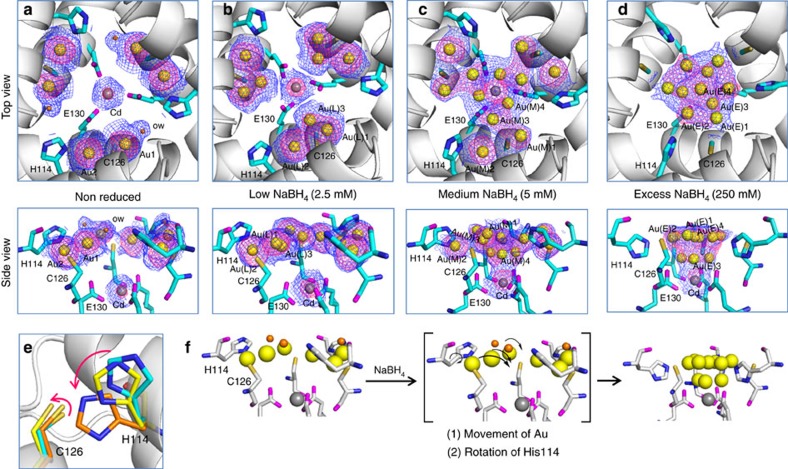
Process of formation of gold sub-nanocluster with evidence from crystal structures. (**a**–**d**) Crystal structures showing the Au nucleation process at the three-fold channel of apo-ferritin cage. The selected 2*F*_o_*-F*_c_ maps at 1*σ* and anomalous difference Fourier density maps at 4*σ* are shown in blue and pink colours, respectively. (**e**) is showing the conformational changes of His114 and Cys126 during the reduction process. Cyan: before reduction; yellow: medium NaBH_4_; orange: excess NaBH_4_. (**f**) Proposed mechanism of the gold sub-nanocluster formation at the three-fold channel of apo-E45C/R52C-rHLFr. The yellow spheres are for Au atoms and the orange spheres are for water molecules.

After observing formation of the Au sub-nanocluster at the three-fold axis channel, we investigated the movement of Au ions by determining crystal structures at different stages of Au reduction. We reduced the Au·CL-apo-E45C/R52C-rHLFr crystals by soaking into low (2.5 mM) and medium (5 mM) concentrations of NaBH_4_ for 1 h, which are represented as Au^0^(L)·CL-apo-E45C/R52C-rHLFr and Au^0^(M)·CL-apo-E45C/R52C-rHLFr, respectively, and measured the X-ray diffractions. Selected crystallographic parameters are included in the supporting information (Supplementary Tables 6–9). At the initial stage of reduction, when a low concentration of NaBH_4_ was used, the anomalous electron density map of Au atoms in Au^0^(L)·CL-apo-E45C/R52C-rHLFr remains similar to that of Au·CL-apo-E45C/R52C-rHLFr, except that Au(L)1 is coordinated by Cys126 and a water molecule (Fig. 4a,b). The deformed anomalous density map of Au(L)1 indicates initial movement of the Au(L)1 atom in Au^0^(L)·CL-apo-E45C/R52C-rHLFr. An extra Au atom (Au(L)3) was placed in the elongated anomalous map to show the movement of Au(L)1 (Fig. 4b). When an intermediate concentration of NaBH_4_ (5 mM) is used, the anomalous electron density map of Au(M)1 in Au^0^(M)·CL-apo-E45C/R52C-rHLFr becomes more extended, clearly indicating movement of Au1 of Au·CL-apo-E45C/R52C-rHLFr towards the axis centre for clustering (Fig. 4a,c). Interestingly, during this process, the Au(M)2 atom coordinated by His114 and Cys126 remains in the same position, suggesting that the Au(M)1 atom, which is coordinated by only a single amino-acid residue (Cys126), moves first and then Au(M)2 starts moving (Fig. 4c). The extended anomalous density map of Au(M)1 also gives an indication about the initial formation of trinuclear clusters (Fig. 4c)^47^,^48^. The map suggests that the Au(M)1 is involved in forming the bottom layer of the final sub-nanocluster (Fig. 4c,d, side view). When excess amounts of NaBH_4_ are used, the anomalous map in Au^0^(E)·CL-apo-E45C/R52C-rHLFr indicates complete movement of both Au(M)1 and Au(M)2 to form the sub-nanocluster (Fig. 4c,d). During the entire reduction process, it was observed that the conformations of His114 and Cys126 at the three-fold axis channel are altered and become exposed towards the centre of the axis channel to stabilize the sub-nanocluster (Fig. 4e). His114 was found to be largely involved in stabilizing the sub-nanocluster. The reduction of Au1 in Au·CL-apo-E45C/R52C-rHLFr causes disruption of Au1-O_ow_ bonds and the reduction of Au2 causes conformational changes of His114 and Cys126 (Fig. 4a,b,e). During the formation of the sub-nanocluster at the three-fold axis channel, we observed disappearance of Au ions Au4, Au6, Au7 and Au8 from other binding sites at the metal accumulation centre and Met96 binding site (Supplementary Fig. 4). These Au atoms might be participating in formation of the sub-nanocluster at the three-fold channel. Thus, the crystal structures obtained during different stages of Au reduction demonstrate that the formation of Au sub-nanoclusters occurs in two steps: (1) movement of Au ions and (2) changing of His conformations (Fig. 4f). The present data show only the detail about the formation of gold sub-nanocluster with a series of X-ray crystal structures, although the Au sub-nanoclusters formed in the ferritin cage are expected to form larger structures as previously described in the literatures^47^,^49^,^50^.

## Discussion

The results of the X-ray crystal structure analyses suggest that a Au sub-nanocluster is formed in the protein scaffold. The crosslinked crystalline protein cage is sufficiently stable to maintain its lattice structure and the amino-acid residues remain available to stabilize the metal coordination structures. This allowed us to study the chemical reduction reaction of gold inside the protein cage crystal and to observe the movement of Au ions during formation of the sub-nanocluster. Previously we determined the pre-organization of Pd ions/complexes on the interior surface of ferritin cage and their mechanism of accumulation^16^,^33^. Here, we showed the determination of the nucleation process of Au ions within the single crystal of ferritin cage under reduction condition because decomposition of the single crystal in the presence of strong reducing agent (NaBH_4_) was prevented by crosslink treatment. Cys126 and Cys48 are found to be the common binding sites of Au ions and Pd ions/complexes, although their coordination structures are unique (Supplementary Fig. 5). The Au ions bridged by Cys126 form unique linear coordination structures with either His114 or a water molecule, giving S^γ^(Cys126)-Au2-N^ɛ^(His114) angle of 173° and S^γ^(Cys126)-Au1-O_ow_ of 170°, respectively. Similarly, at the metal accumulation centre, the thiol-(Cys48, Cys52) bridged trinuclear Au structure has three linear units, giving S^γ^(Cys52)-Au4-O_ow_, S^γ^(Cys52)-Au3-S^γ^(Cys48) and S^γ^(Cys48)-Au5-N^ɛ^(His49) angle of 177°, 171° and 164°, respectively. On the other hand, Pd ions/complexes bridged by either Cys126 or Cys48 do not form linear coordination structures. Additionally, the Pd ions have binding preferences towards Asp40, Glu45and Arg52, whereas the Au ions specifically bind at Cys48, Cys126 and Met96. Previously we observed the formation of a trinuclear Pd(II) structure at the three-fold symmetric channel when the His114 was replaced by Ala^34^. This suggests that the symmetric three-fold axis channel of the ferritin cage is the suitable position for studying the nucleation and formation of the Au sub-nanocluster because of the unique coordination environment generated by the amino-acid residues from three different monomeric subunits, which are directed towards the axis centre. As such, this position does not require additional stabilizing ligands. There are three important factors that significantly influence the formation of the Au sub-nanocluster. First, the restricted size of the three-fold symmetric cavity provides a suitable coordination environment and induces the pre-organized Au ions from monomeric subunits to approach each other to form the sub-nanocluster in the presence of a reducing agent. Second, the Cd atom coordinated by Glu130 plays an important role in stabilizing the sub-nanocluster by forming an Au_3_Cd unit as described in the literature (Supplementary Fig. 3)^41^,^42^,^43^. The Cd atom could serve as a seed for Au sub-nanocluster formation in the three-fold channel in a manner similar to that observed for the Fe_3_O_4_ nanocluster in the Dps-like (Dps, DNA-protecting protein during starvation) ferritin protein DpsA^10^. Last, the flexibility of the amino-acid residues influences the formation of the sub-nanocluster, which was evidenced from the conformational changes during the reduction of Au ions (Fig. 4e). His114 was found to play a major role in stabilizing the sub-nanocluster as it has affinity towards both Au^0^ and Au ions. His114 is flexible enough to adopt different metal coordination structures^51^,^52^,^53^. The flexible Glu130 also contributes to stabilization of the Au sub-nanocluster by capturing one Cd atom (Fig. 4a–d).

It is suggested that nanoclusters form the initial metal core or nucleation site for nanoparticle formation. For example, cobalt oxide nanoparticles are fabricated in the ferritin cage and an Fe_3_O_4_ nanocluster is formed in Dps-like ferritin^10^,^18^. The metal oxide/chloride cluster in the symmetric protein scaffold does not grow to generate a larger mineral and thus it can serve as an electron transfer centre^12^,^13^. Several organic ligands have been developed to stabilize small nanoclusters and crystal structures have been obtained, but the formation mechanism remains unknown^46^,^49^,^50^. Similarly, protein scaffolds are also used for preparing small nanoclusters, but their exact positions, structures and formation mechanisms remain unclear^10^,^11^,^12^,^13^. In this regard, the present work provides a detailed map for studying the mechanism of formation of inorganic materials in the confined protein environment. So, it is expected that the results of the present work will provide insights for design of protein scaffolds by suitable amino-acid replacements for preparation and stabilization of small nanoclusters, which are important for catalysis and bioimaging.

Interestingly, we were able to observe the sub-nanocluster formation only when the Au ions were reduced inside the crystalline protein cage. We attempted to observe such Au sub-nanocluster formation by reducing Au·apo-E45C/R52C-rHLFr with 5,000 equiv of NaBH_4_ in solution followed by purification and crystallization. Although we observed a weak surface plasmon absorbance near 520 nm, we failed to observe sub-nanocluster formation in the X-ray crystal structure analysis (Supplementary Fig. 6). When the same reaction proceeded in the presence of Cd ions in solution, we failed to crystalize the composite. One possibility is that increased structural flexibility and fast reaction kinetics in solution compared to crystalline condition prevent the sub-nanocluster from being observed. The results show the advantage of using a crystalline protein cage for studying the protein-nanostructure interactions, for investigating the mechanism of various chemical reactions and biomineralization processes and for developing solid biohybrid materials^19^,^21^,^54^. In addition to investigating the apo-E45C/R52C-rHLFr mutant, we also examined wild-type apo-rHLFr and observed similar conformational changes of amino-acid residues (His114 and Cys126) at the three-fold axis channel; however, the anomalous density at 4*σ* was found to be too weak to model the clustering site. This is possibly due to less efficient uptake of Au ions.

In summary, we have demonstrated structural evidence for the formation of an Au sub-nanocluster inside the narrow three-fold symmetric channel of the apo-ferritin cage. Gradual movement of the Au ions during the reduction reaction was directly visualized from the X-ray crystal structure analysis. The results show how the Au sub-nanoclusters interact with the protein environment and the dynamics of the amino-acid residues during their formation. The main conclusions of the work are as follows: First, since small clusters are believed to form the initial core during assembly of metal nanoparticles, our findings are expected to provide a useful platform for future exploration of the molecular mechanism of biomineralization and nanoparticle growth in biomolecular scaffolds. Second, the observation of formation of a sub-nanocluster within the symmetric environment provides an important aspect for designing protein pocket for small clusters, which are difficult to synthesize and stabilize due to aggregation. The detailed mechanism of sub-nanocluster formation is being further investigated by suitable amino-acid modification at the three-fold symmetric channel, which could provide the additional stabilization. Such small clusters are considered to be the most desirable for use as catalysts and bioimaging agents. Finally, the crosslinking of protein crystals could be useful to maintain their lattice structure under harsh conditions. The present work is expected to expand the scope of studying molecular mechanisms by promoting chemical reactions inside various crosslinked protein crystals and developing solid materials.

## Methods

### Materials

All reagents were purchased from commercial suppliers like TCI, Wako, Nacalai Tesque and Sigma–Aldrich and used as received. Recombinant L-chain apo-rHLFr from horse liver (rHLFr) was prepared in NovaBlue competent cells (Novagen) transformed with the expression vector pMK2. The culture and purification of apo-Ferritin was carried out by following the previous report^55^. Apo-E45C/R52C-rHLFr mutant was prepared using a Stratagene Quikchange MultiSite kit. The UV-visible absorption spectral measurements were performed using UV-2400PC UV−vis spectrometer (Shimazu). Au concentrations in Au·apo-E45C/R52C-rHLFr were determined by ICP-MS (PerkinElmer, Elan DRC-e instrument). Standard curves for Au atom were obtained by using Au standard solution (20–1,000 p.p.m.).

### Preparation of Au·apo-E45C/R52C-rHLFr

The preparation of the Au·apo-E45C/R52C-rHLFr was done by following our previously reported procedure^36^. An aqueous solution of apo-E45C/R52C-rHLFr in 0.15 M NaCl (2 μM, 15 ml) was adjusted to pH 8.5 with 10 mM NaOH, followed by the addition of aliquots of KAuCl_4_ aqueous solution (200 equiv., 0.4 mM). Then, the mixture was stirred for 30 min at RT followed by dialysis against 0.15 M NaCl to remove any unbound Au ions. The dialysed solution was filtered and purified using a gel filtration column (Sephadex G25) equilibrated with 0.15 M NaCl. The concentration of Au atoms and protein were determined by ICP-MS and BCA assay, respectively.

### Crystallization

The crystallization of Au·apo-E45C/R52C-rHLFr was performed by the hanging-drop vapour diffusion method^16^,^34^. The drops were prepared by mixing an equal volume (1.5 μl) of concentrated protein solution (20–30 mg ml^−1^) and the precipitant solution (0.5–1 M (NH_4_)_2_SO_4_, 10–20 mM CdSO_4_) and equilibrated against the precipitant solution (1.0 ml) at 20 °C. The crystals were obtained within a day.

### Crosslink treatment and chemical reaction inside crystal

Freshly prepared crystals (within 1 day) were used to minimize the auto-reduction of Au ions, which gives light blue/purple colour. The crystals were first washed with a precipitant solution containing 0.5 M Na_2_SO_4_ and 20 mM CdSO_4_. Then, the crystals were soaked into a solution containing 0.5 M Na_2_SO_4_, 20 mM CdSO_4_ and 1% glutaraldehyde for 3 h at 20 °C. The crosslinked crystals were then washed with a solution containing 0.5 M Na_2_SO_4_. Reduction of the Au ions inside the crystals was carried out by soaking into a NaBH_4_ solution containing 0.5 M Na_2_SO_4_ and 10 mM Tris-HCl (pH 8.0) for 1 h at 20 °C. During this process, the colour of the crystals became dark brown. After reduction, the crystals were washed (4–5 times) with the precipitant solution containing 0.5 M Na_2_SO_4_ and 10 mM Tris-HCl (pH 8.0). Finally, the crystals were stored in a solution containing 0.5 M Na_2_SO_4_, 20 mM CdSO_4_ and 10 mM Tris-HCl before SPring-8 data collection.

### X-ray data collection

The X-ray diffraction data of the ferritin crystals containing Au were collected at beam line BL38B1 or BL26B1 (SPring-8). Before data collection, crystals were soaked into the precipitant solution (0.5 M Na_2_SO_4_, 20 mM CdSO_4_ and 10 mM Tris-HCl) containing 25% (w/w) ethylene glycol and subsequently frozen in liquid nitrogen. X-ray diffraction data were collected at 100 K using X-ray wavelengths of 1.03 and 1.05 Å, which represent the peak and remote wavelengths of Au X-ray absorption, respectively. The data were processed using HKL2000 programs in the cubic *F*432 space group. Selected crystallographic parameters are summarized in Supplementary Tables 1, 4, 6 and 8.

### Refinement

The crystal structures of the Au-ferritin composites were determined by molecular replacement method (MOLREP) using the apo-rHLFr structure (pdbID: 1DAT) as initial model. The structures were refined by using REFMAC5 in CCP4 suit and re-built in COOT using sigma-weighted (2*F*_o_-*F*_c_) and (*F*_o_-*F*_c_) electron-density maps. Water molecules were positioned to fit residual (*F*_o_-*F*_c_) density peaks with a lower cutoff of 3*σ*. The positions of the Au atoms were determined from the observed anomalous difference Fourier maps with a lower cutoff of 4*σ*. The Cd binding sites were assigned by comparing previous reported structures^16^,^36^,^37^. The Au atoms were distinguished from Cd atoms by comparison with the control ferritin structure as well as the anomalous density at Au peak (1.03 Å) and remote (1.05 Å) wavelengths. The occupancy values of Au and Cd atoms were adjusted manually by considering the surrounding negative density and refined with fractional occupancy (Supplementary Tables 2,5,7 and 9). The *B*-factors of the Au atoms are in the range of 26–56 Å^2^, except that the Au atoms at the three-fold channels of the structures Au^0^(M)·CL-apo-E45C/R52C-rHLFr and Au^0^(E)·CL-apo-E45C/R52C-rHLFr, which are in the range of 77–96 Å^2^. Ser1, Ser2, His173 and Asp174 of all the structures and Lys172 for the structures Au^0^(M)·CL-apo-E45C/R52C-rHLFr and Au^0^(E)·CL-apo-E45C/R52C-rHLFr were not decided due to disordered electron density. Arg18, Asp135, Ser157 and Lys172 of Au·CL-apo-E45C/R52C-rHLFr; Lys97 and Lys172 of Au^0^(L)·CL-apo-E45C/R52C-rHLFr; Arg18, Glu53, Glu56, Ser131, Asp135, Ser157, Gln158 of Au^0^(M)·CL-apo-E45C/R52C-rHLFr; and His49 and Ser157 of Au^0^(E)·CL-apo-E45C/R52C-rHLFr were replaced by Ala due to low electron density of the side chain. The models were subjected to quality analysis during the various refinement stages with omit maps and RAMPAGE. Two experiments were performed separately to test the reproducibility. Atomic coordinates of the crystal structures of Au·CL-apo-E45C/R52C-rHLFr, Au^0^(L)·CL-apo-E45C/R52C-rHLFr, Au^0^(M)·CL-apo-E45C/R52C-rHLFr and Au^0^(E)·CL-apo-E45C/R52C-rHLFr were deposited in the Protein Data Bank under accession codes of 5GU0, 5GU1, 5GU2 and 5GU3, respectively.

### Data availability

Crystallographic data table, additional figures, tables and comparisons are available in the Supplementary Information files. All the crystal structures are deposited at the Protein Data Bank and can be accessed with deposition codes 5GU0, 5GU1, 5GU2 and 5GU3.

## Additional information

**How to cite this article:** Maity, B. *et al*. Observation of gold sub-nanocluster nucleation within a crystalline protein cage. *Nat. Commun.* **8,** 14820 doi: 10.1038/ncomms14820 (2017).

**Publisher's note**: Springer Nature remains neutral with regard to jurisdictional claims in published maps and institutional affiliations.

## Supplementary Material

Supplementary Information Supplementary Figures, Supplementary Tables and Supplementary References.
