# Supplementary material for: Observation of gold sub-nanocluster nucleation within a crystalline protein cage
Source: Nat Commun. 2017 Mar 16;8:14820. doi: 10.1038/ncomms14820 (PMC5357307; doi:10.1038/ncomms14820)
Supplement: Supplementary Information — Supplementary Figures, Supplementary Tables and Supplementary References. [file ncomms14820-s1.pdf]

Supplementary Table 1. Summary of X-ray data collection and refinement statistics for **Au•CL-apo-E45C/R52C-rHLFr**.

	**Au•CL-apo-E45C/R52C-rHLFr**	
	Au Peak	Au Remote
**Data collection**		
X-ray wavelength (Å)	1.03586	1.05777
Space group	*F*432	*F*432
Cell dimentions		
a = b = c (Å)	180.89	180.97
α = β = γ (°)	90	90
Resolution (Å)	40-1.95	50-1.95
	(1.98-1.95)	(1.98-1.95)
R_{meas} (%)	7.8 (29.2)	7.5 (28.2)
$I/\sigma(I)$	65.7 (12.8)	67.8 (12.7)
Completeness (%)	99.9 (100)	99.9 (100)
Redundancy	11.0 (11.3)	11.1 (11.3)
**Refinement**		
Resolution (Å)	1.95	
No. of reflections	18,055	
R_{work}/R_{free}	0.177 / 0.204	
No. of residues	170	
No. of water molecules	77	
No. of Au atoms	8	
No. of Cd atoms	3	
No. of sulfate ions	2	
No. of ethylene glycol	2	
Average *B*-factor (Å^2)	29.49	
r. m. s. deviation		
bond lengths (Å)	0.0195	
bond angles (°)	1.9395	
Ramachandran plot (%)		
most favored	98.2	
allowed	1.8	
outlier	0	

Values in parentheses are for the highest-resolution shell.

Supplementary Table 2. B-Factors (B.F.) and occupancies (Occu.) of metal atoms in **Au•CL-apo-E45C/R52C-rHLFr**.

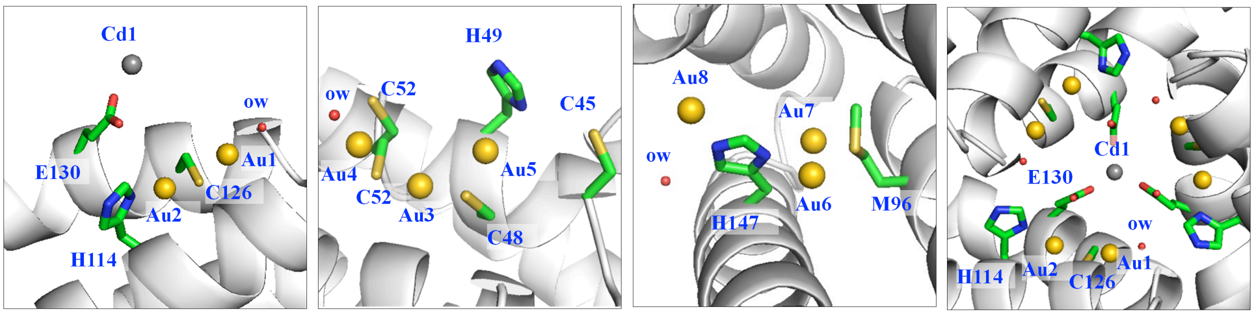

Site-A Site-B Site-C 3-Fold channel

**Atom**	**Au1**	**Au2**	**Au3**	**Au4**	**Au5**	**Au6**	**Au7**	**Au8**	**Cd1**
**B.F.**	55.78	46.10	28.79	42.50	42.50	35.00	25.99	43.92	40.37
**Occu.**	0.55	0.55	0.55	0.50	0.50	0.3	0.5	0.4	0.20

Supplementary Table 3. Bond distances of Au atoms with adjacent amino acids in **Au•CL-apo-E45C/R52C-rHLFr**.

**Bond**	**Bond distance (Å)**	**Bond**	**Bond distance (Å)**
Au1-O_{ow}	2.39	Au4- S^{γ} (Cys48)	2.19
Au1-S^{γ} (Cys126)	2.39	Au4- N^{ε} (His49)	2.74
Au2- S^{γ} (Cys126)	2.26	Au6- N^{ε} (His147)	2.33
Au2- N^{ε} (His114)	2.32	Au6- S^{ε} (Met96)	3.09
Au3- S^{γ} (Cys48)	2.19	Au7- N^{ε} (His147)	2.44
Au3- S^{γ} (Cys52)	2.22	Au7- S^{ε} (Met96)	1.89
Au5- S^{γ} (Cys52)	2.31	Au8- N^{ε} (His147)	2.15
Au5-O_{ow}	2.35		

Supplementary Table 4. Summary of X-ray data collection and refinement statistics for **Au^0(E)•CL-apo-E45C/R52C-rHLFr** .

	**Au^0(E)•apo-E45C/R52C-rHLFr**	
	Au Peak	Au Remote
**Data collection**		
X-ray wavelength (Å)	1.03586	1.05777
Space group	*F*432	*F*432
Cell dimentions		
a = b = c (Å)	181.50	181.53
α = β = γ (°)	90	90
Resolution (Å)	40-2.03	30-2.03
	(2.07-2.03)	(2.06-2.03)
R_{meas} (%)	8.0 (31.4)	7.7 (29.2)
$I/\sigma (I)$	63.7 (11.5)	66.4 (12.4)
Completeness (%)	99.9 (100)	99.9 (100)
Redundancy	10.9 (11.2)	10.9 (11.2)
**Refinement**		
Resolution (Å)	2.03	
No. of reflections	16,062	
R_{work}/R_{free}	0.189 / 0.244	
No. of residues	169	
No. of water molecules	84	
No. of Au atoms	6	
No. of Cd atoms	5	
No. of ethylene glycol	3	
No. of sulfate ions	2	
Average *B*-factor ($Å^2$)	33.26	
r. m. s. deviation		
bond lengths (Å)	0.0199	
bond angles (°)	1.8411	
Ramachandran plot (%)		
most favored	97	
allowed	3	
outlier	0	

Values in parentheses are for the highest-resolution shell.

Supplementary Table 5. B-Factors (B.F.) and occupancies (Occu.) of metal atoms in **Au^0(E)•CL-apo-E45C/R52C-rHLFr**.

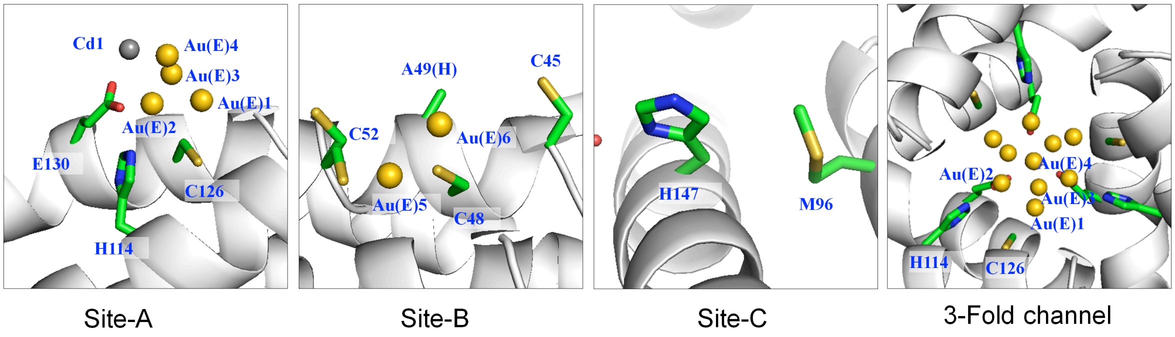

Atoms	Au(E)1	Au(E)2	Au(E)3	Au(E)4	Au(E)5	Au(E)6	Cd1
**B.F.**	91.05	95.95	89.13	89.71	33.86	50.43	54.39
**Occu.**	0.20	0.20	0.25	0.15	0.35	0.30	0.20

Supplementary Table 6. Summary of X-ray data collection and refinement statistics for **Au^0(L)•CL-apo-E45C/R52C-rHLFr.**

	**Au^0(L)•CL-apo-E45C/R52C-rHLFr**	
	Au Peak	Au Remote
**Data collection**		
X-ray wavelength (Å)	1.0385	1.0578
Space group	*F*432	*F*432
Cell dimentions		
a = b = c (Å)	181.17	181.19
α = β = γ (°)	90	90
Resolution (Å)	40-2.05 (2.09-2.05)	42-2.05 (2.09-2.05)
R_{meas} (%)	7.2 (31.0)	7.0 (28.3)
$I/\sigma(I)$	63.2 (11.5)	66.3 (12.6)
Completeness (%)	99.9 (100)	99.9 (100)
Redundancy	11.0 (11.3)	11.0 (11.03)
**Refinement**		
Resolution (Å)	2.05	
No. of reflections	15,665	
R_{work}/R_{free}	0.185 / 0.223	
No. of residues	170	
No. of water molecules	80	
No. of Au atoms	9	
No. of Cd atoms	8	
No. of sulfate ions	2	
No. of ethylene glycol	2	
Average *B*-factor ($Å^2$)	31.75	
r. m. s. deviation		
bond lengths (Å)	0.0189	
bond angles (°)	1.8238	
Ramachandran plot (%)		
most favored	97.6	
allowed	2.4	
outlier	0	

Values in parentheses are for the highest-resolution shell.

Supplementary Table 7. B-Factors (B.F.) and occupancies (Occu.) of metal atoms in **Au^0(L)•CL-apo-E45C/R52C-rHLFr**.

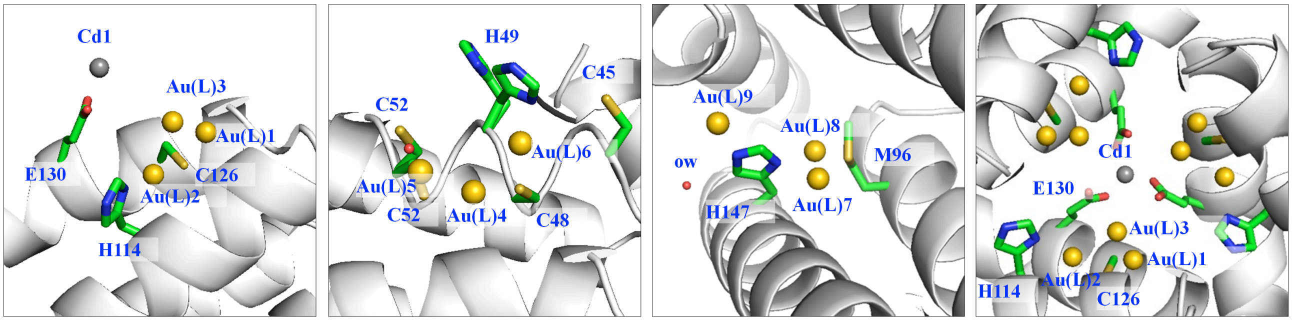

Site-A Site-B Site-C 3-Fold channel

**Atoms**	**Au(L)1**	**Au(L)2**	**Au(L)3**	**Au(L)4**	**Au(L)5**	**Au(L)6**	**Au(L)7**	**Au(L)8**	**Au(L)9**	**Cd1**
**B.F.**	49.99	47.51	44.00	33.00	51.63	46.59	35.70	26.44	42.42	35.82
**Occu.**	0.40	0.55	0.20	0.55	0.50	0.50	0.30	0.50	0.30	0.25

Supplementary Table 8. Summary of X-ray data collection and refinement statistics for **Au^0(M)•CL-apo-E45C/R52C-rHLFr.**

	**Au^0(M)•CL-apo-E45C/R52C-rHLFr**
	Au Peak
**Data collection**	
X-ray wavelength (Å)	1.03895
Space group	*F*432
Cell dimentions	
a = b = c (Å)	181.08
α = β = γ (°)	90
Resolution (Å)	30-2.12
	(2.16-2.12)
R_{meas} (%)	6.3 (30.6)
$I/\sigma(I)$	60.7 (11.1)
Completeness (%)	99.9 (100)
Redundancy	11.5 (11.5)
**Refinement**	
Resolution (Å)	2.12
No. of reflections	14,079
R_{work}/R_{free}	0.185 / 0.229
No. of residues	169
No. of water molecules	47
No. of Au atoms	10
No. of Cd atoms	6
No. of ethylene glycol	1
No. of sulfate ions	2
Average *B*-factor ($Å^2$)	36.17
r. m. s. deviation	
bond lengths (Å)	0.0198
bond angles (°)	1.8450
Ramachandran plot (%)	
most favored	97
allowed	3
outlier	0

Values in parentheses are for the highest-resolution shell.

Supplementary Table 9. B-Factors (B.F.) and occupancies (Occu.) of metal atoms in **Au^{0}(M)•CL-apo-E45C/R52C-rHLFr**.

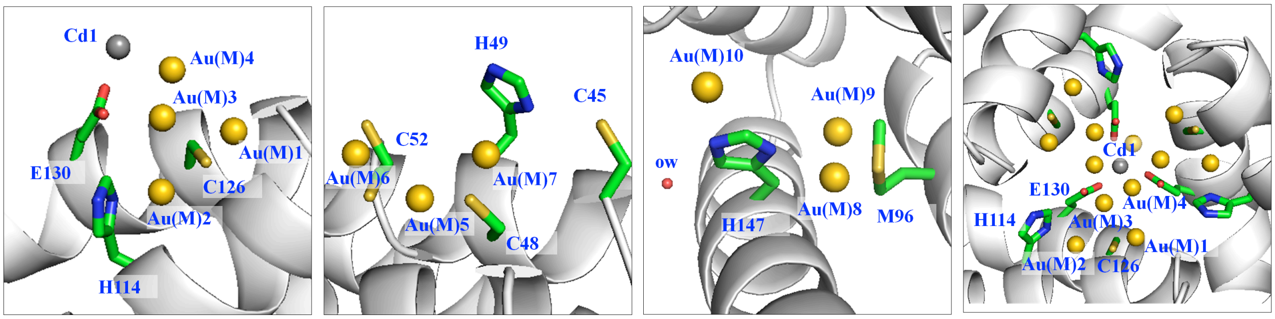

Site-A Site-B Site-C 3-Fold channel

Atoms	Au(M)1	Au(M)2	Au(M)3	Au(M)4	Au(M)5	Au(M)6	Au(M)7	Au(M)8	Au(M)9	Au(M)10	Cd1
**B.F.**	89.10	66.50	76.63	82.15	38.39	44.76	52.63	45.70	44.59	54.09	50.17
**Occu.**	0.30	0.40	0.20	0.20	0.60	0.15	0.45	0.15	0.25	0.15	0.25

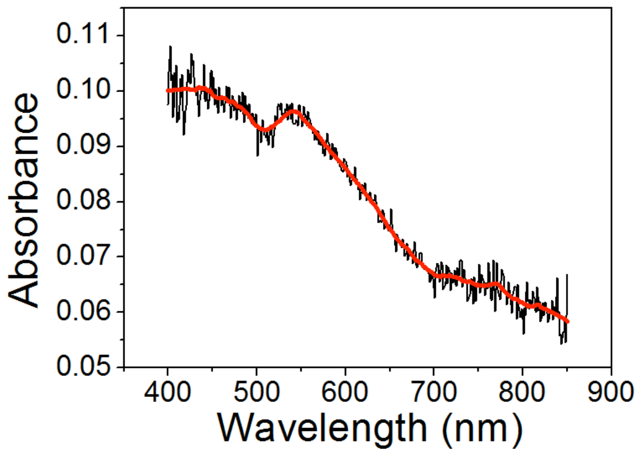

Supplementary Figure 1: Absorption spectrum of the crystals of **Au^{0}(E)•CL-apo-E45C/R52C-rHLFr** which were dispersed in aqueous 80% glycerol solution.

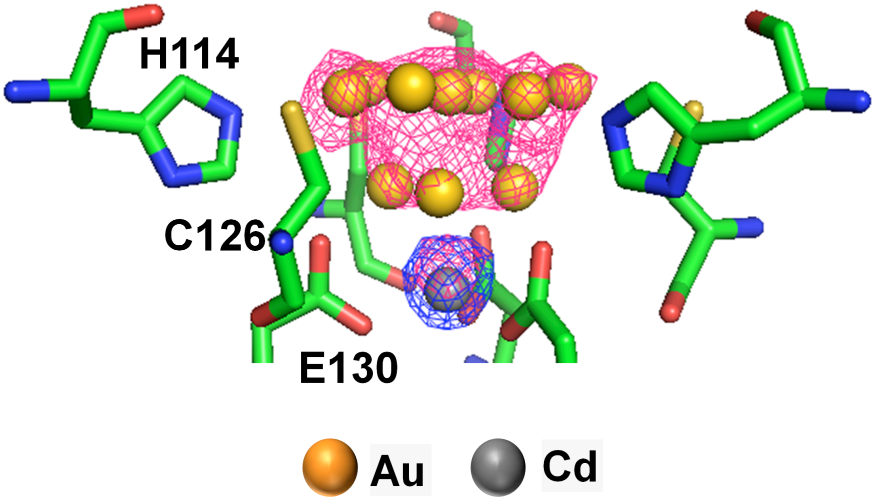

Supplementary Figure 2: Overlap of the anomalous density maps (4σ) for the wavelengths 1.035 Å (peak) and 1.057 Å (remote) at the 3-fold channel of **Au^{0}(E)•CL-apo-E45C/R52C-rHLFr** to distinguish the Au atoms from Cd. Pink map: 1.035Å ; Blue map: 1.057Å.

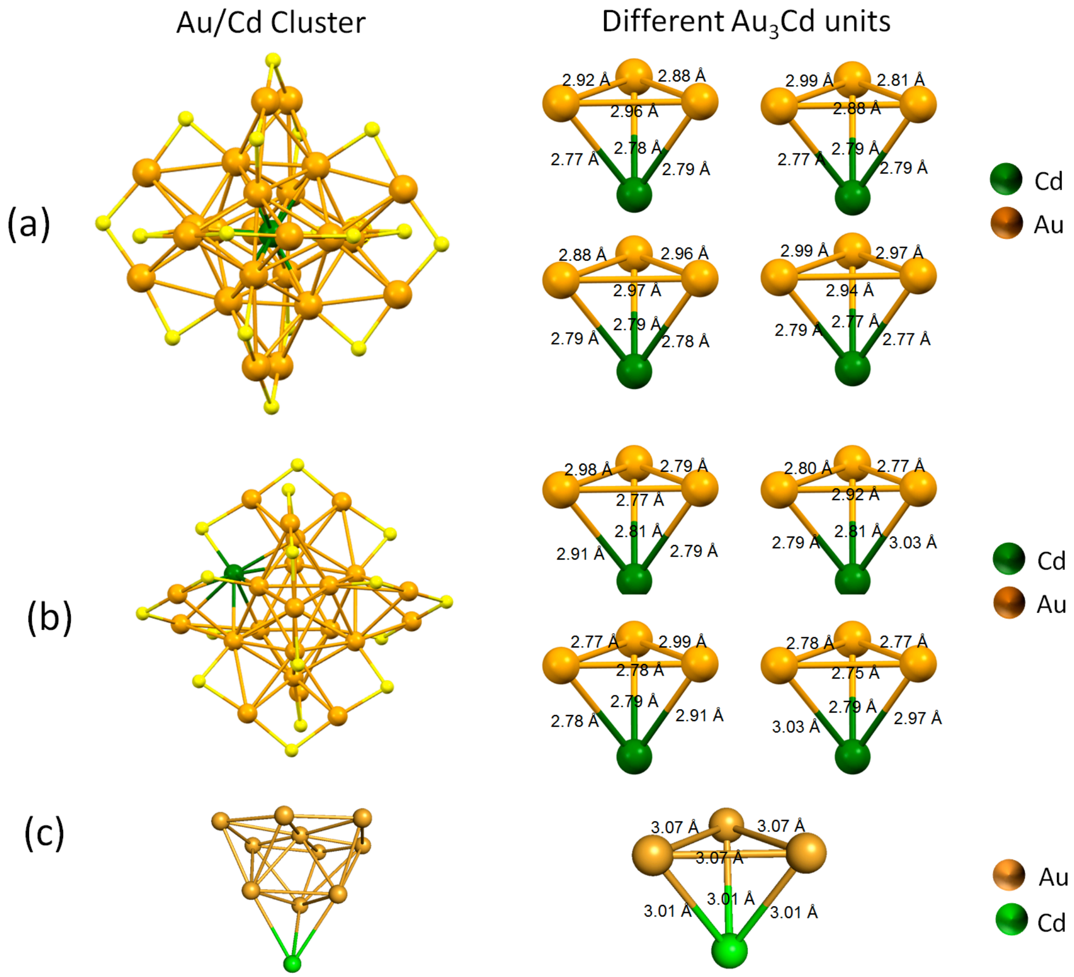

Supplementary Figure 3: Comparison of the Au_3Cd unit present in the 3-fold channel of **Au^0(E)•CL-apo-E45C/R52C-rHLFr** with structures of various Au_3Cd units present in the previously reported Au/Cd nanoclusters.[1, 2] (a) Total structure of $Au_{24}Cd(PhC_2H_4S)_{18}$ and various Au_3Cd units present in the nanocluster.[1] (b) Total structure of $Au_{24}Cd(PhC_2H_4S)_{18}$ and various Au_3Cd units present in the nanocluster.[2] (c) Au/Cd nanocluster formed in the 3-fold axis channel of ferritin cage and the Au_3Cd unit. Au atoms are shown in orange sphere and the Cd atoms are shown in green sphere.

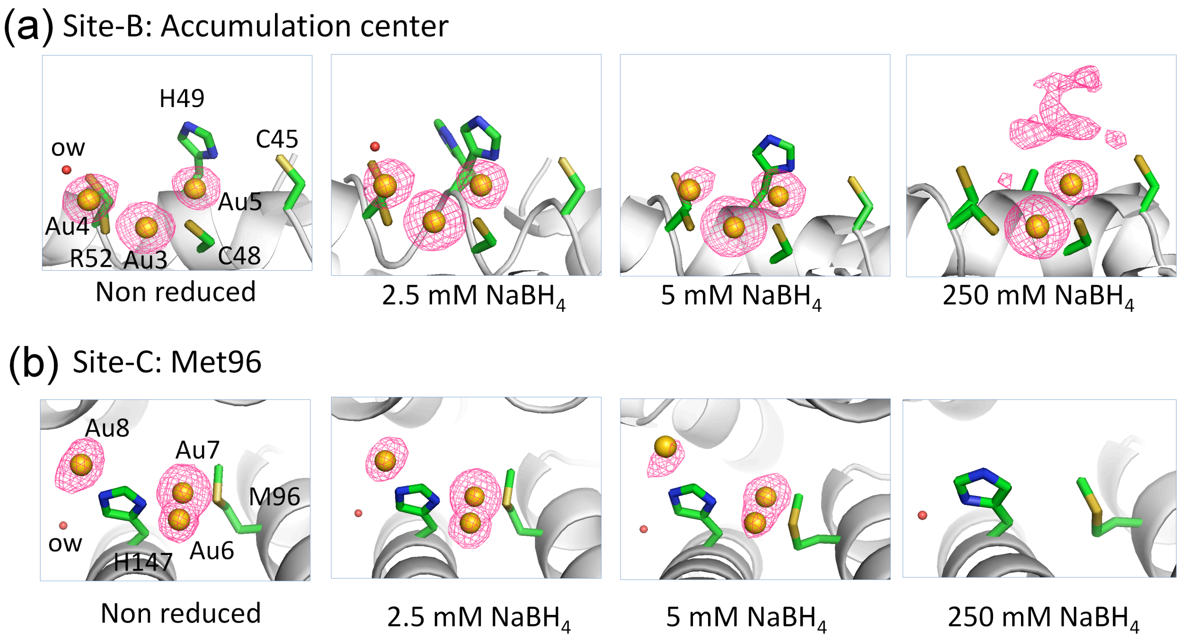

Supplementary Figure 4: Changes of the anomalous density maps of Au atoms of **Au-CL.apo-E45C/R52C-rHLFr** (non-reduced) after the reduction with various concentrations of $NaBH_4$. (a) is showing the anomalous density maps of Au atoms at accumulation center (Site-B). (b) is showing the anomalous density maps of Au atoms at Met96 binding center (Site-C). The anomalous difference Fourier maps at 4σ level are shown in pink.

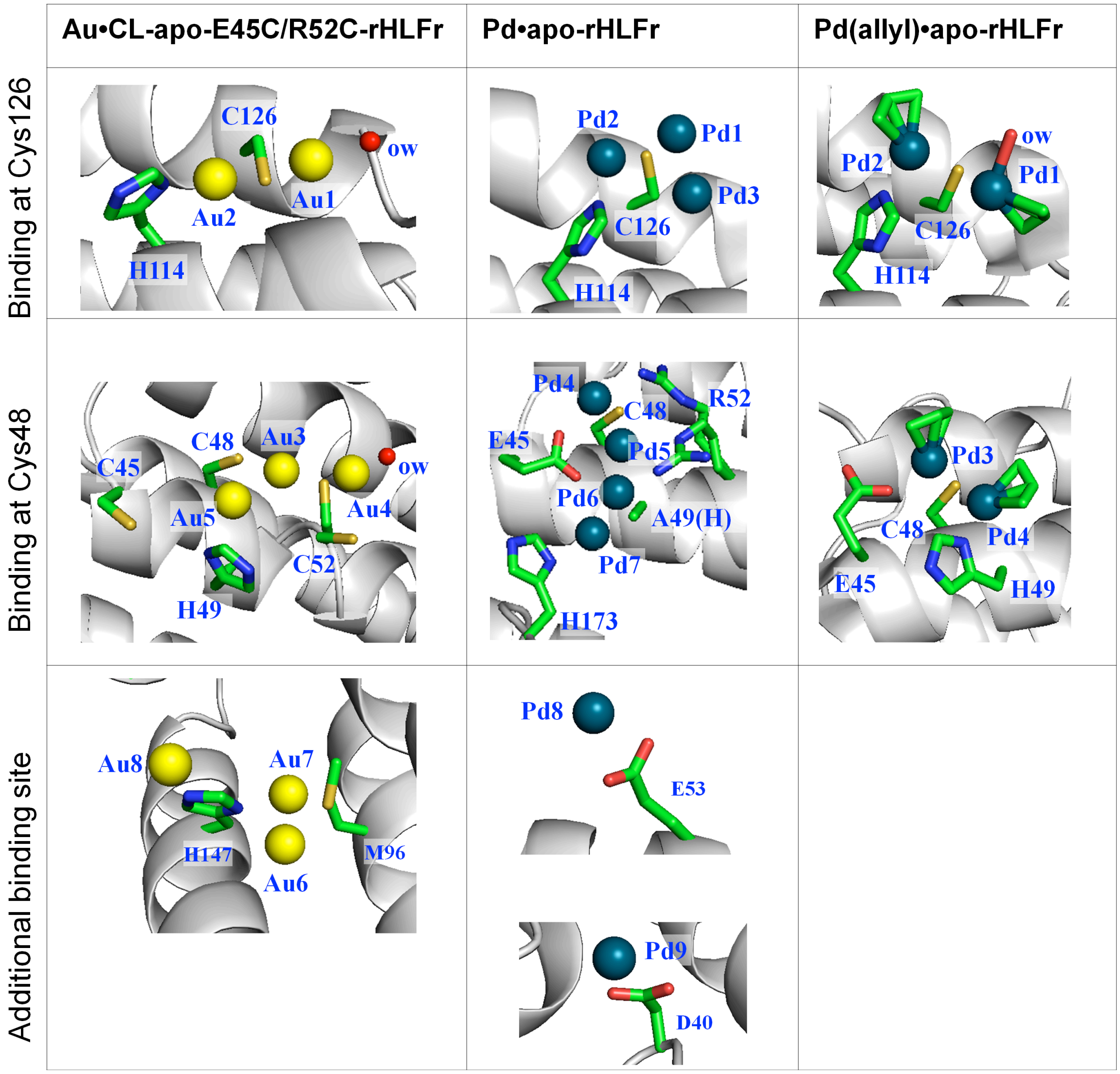

Supplementary Figure 5: Comparison of the coordination structures of Au ions, Pd ions and Pd(allyl) complexes in **Au•CL-apo-E45C/R52C-rHLFr**, **Pd•apo-rHLFr** and **Pd(allyl)•apo-rHLFr**, respectively.[3,4] Au and Pd atoms are shown in yellow and blue spheres, respectively.

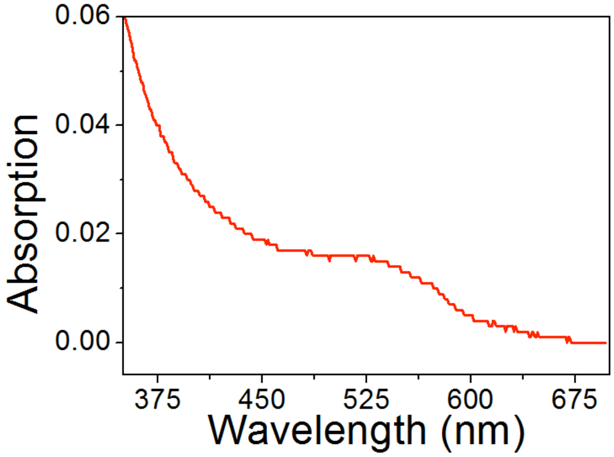

Supplementary Figure 6: Absorption spectrum of the solution of **Au•apo-E45C/R52C-rHLFr** in 0.15M NaCl which was reduced with $NaBH_4$ in solution**.**

Supplementary references

1. Yao, C. et al. Mono-cadmium vs mono-mercury doping of Au_{25} nanoclusters. *J. Am. Chem. Soc.* **137***,* 15350-15353 (2015).
2. Wang, S. et al. Metal exchange method using Au_{25} nanoclusters as templates for alloy nanoclusters with atomic precision. *J. Am. Chem. Soc.* **137***,* 4018-4021 (2015).
3. Ueno, T. et al. Process of accumulation of metal ions on the interior surface of apo-ferritin: crystal structures of a series of apo-ferritins containing variable quantities of Pd(II) ions. *J. Am. Chem. Soc.* **131**, 5094-5100 (2009).
4. Abe, S., Hikage, T., Watanabe, Y., Kitagawa, S. & Ueno, T. Mechanism of accumulation and incorporation of organometallic Pd complexes into the protein nanocage of apo-ferritin. *Inorg. Chem.* **49**, 6967-6973 (2010).
